# Blood oxygen level dependent and adenosine-perfusion imaging correlates to invasive measurement of fractional flow reserve

**DOI:** 10.1186/1532-429X-14-S1-P300

**Published:** 2012-02-01

**Authors:** Peter Bernhardt, Robert Manzke, Thomas Walcher, Wolfgang Rottbauer

**Affiliations:** 1 University of Ulm, Ulm, Germany; 2 Philips Clinical Sites Research Europe, Hamburg, Germany

## Background

It has been shown that blood oxygen level dependent (BOLD) cardiac magnetic resonance imaging (CMR) is able to detect myocardial perfusion differences. However, validation of BOLD CMR against myocardial perfusion reserve (MPR) and fractional flow reserve (FFR) is lacking. We sought to analyze the potential diagnostic accuracy of BOLD CMR in comparison to invasively measured FFR which served as the standard reference.

## Methods

BOLD and contrast-enhanced perfusion image analyses were performed at rest and during adenosine infusion in a 1.5T CMR scanner in three short axes (apical, midventricular and basal), respectively. Thirty-six perfusion territories in twelve patients were analyzed for relative BOLD signal intensity increase and for myocardial perfusion reserve. In all patients invasive FFR measurements were performed in the major three coronary arteries during adenosine infusion. A FFR ≤0.8 was regarded consistent with significant hypoperfusion.

## Results

Relative BOLD signal intensity increase was significantly higher in myocardial segments supplied by coronary arteries with a FFR ≤0.8 (p<0.05). The area under the receiver operator curve for BOLD and MPR analysis did not differ significantly for segments perfused by the left anterior descending artery (0.80 vs. 0.73), left circumflex artery (0.80 vs. 0.98) and right coronary artery (0.97 vs. 0.97).

## Conclusions

CMR BOLD imaging reliably detects hemodynamic significant coronary artery disease and is an alternative to contrast-enhanced perfusion without use of exogenous contrast agents.

## Funding

None.

